# Supplementary material for: Sex differences in association football: a scoping review
Source: PeerJ. 2025 Aug 27;13:e19976. doi: 10.7717/peerj.19976 (PMC12398286; doi:10.7717/peerj.19976)
Supplement: Supplemental Information 1 [file peerj-13-19976-s001.docx]

| PRISMA-ScR Checklist | | |
| --- | --- | --- |
| **Section** | **Item** | **Corresponding sections of the manuscript** |
| **Title** | 1 | Sex differences in elite football players: a scoping review |
| **Abstract** | | |
| Structured summary | 2 | p.1: the ***Abstract*** section of the manuscript. |
| **Introduction** | | |
| Rationale | 3 | p.1-2: All the text that precedes the statement “Therefore, this study is a scoping review of sex differences in elite football players…” within the ***Introduction*** section of the manuscript. |
| Objectives | 4 | p.2: This study is a scoping review of sex differences in elite football players, aiming to: 1) synthesize existing evidence regarding sex differences in elite football players; 2) identify research gaps to provide direction for future studies. |
| **Methods** | | |
| Protocol and registration | 5 | p.2: the ***Study design and protocol registration*** section of the manuscript. |
| Eligibility criteria | 6 | p.3: the ***Eligibility criteria*** section of the manuscript. |
| Information sources | 7 | p.3: The following databases were searched: Scopus, PubMed, ScienceDirect, and Web of Science (Core Collection). These searches encompassed relevant publications available up to 17 October 2024. In addition, manual searches were conducted on the reference lists of the included studies to identify potentially relevant studies. |
| Search | 8 | p.3: Table S1. Full search strategy for each database |
| Selection of sources of evidence | 9 | p.3: This review only included peer-reviewed original research studies in the English language. |
| Data charting process | 10 | p.3: For each study, key information from the included texts was extracted into a custom-designed data extraction form. This form was scrutinized by the research team and pretested by all researchers before implementation to guarantee the accurate collection of information. Two researchers independently extracted data using the form, while a third researcher cross-checked the information to ensure its accuracy and reliability. |
| Data items | 11 | p.3: the ***Data items*** section of the manuscript. |
| Critical appraisal of individual sources of evidence | 12 | p.3: the ***Risk of Bias*** section of the manuscript. |
| Summary measures | 13 | Not applicable for scoping reviews. |
| Synthesis of results | 14 | p.4: the ***Data synthesis*** section of the manuscript. |
| Risk of bias across studies | 15 | p.3: the ***Risk of Bias*** section of the manuscript. |
| Additional analyses | 16 | Not applicable for scoping reviews. |
| **Results** | | |
| Selection of sources of evidence | 17 | p.4: the ***Search Results*** section of the manuscript. |
| Characteristics of sources of evidence | 18 | p.4: the ***Description and Characteristics of the Included Studies*** section; p.5: ***The Usage of Sex and Gender Terminology*** section and the ***Research themes*** of the manuscript. |
| Critical appraisal within sources of evidence | 19 | p.4: the ***Risk of Bias Assessment*** section of the manuscript. |
| Results of individual sources of evidence | 20 | p.5: “The ESM provides a summary of the included studies, which can be accessed via a link available at https://osf.io/gnmky.” |
| Synthesis of results | 21 | p.5: the ***Research Themes*** section of the manuscript. |
| Risk of bias across studies | 22 | p.6: the ***Risk of Bias Assessment*** section of the manuscript. |
| Additional analyses | 23 | Not applicable for scoping reviews. |
| **Discussion** | | |
| Summary of evidence | 24 | p.6-7: “Eighty studies were included, covering 2226 female and 2670 male players, and 99 female and 135 male matches. The literature’s sample size disparity suggests a need for greater focus on female football players. Over 90% of the studies exhibited terminological confusion between ‘sex’ and ‘gender’, indicative of a conceptual ambiguity prevalent in the extant literature. Additionally, the lack of reporting on female players’ menstrual cycles and contraceptive use in 90% of the studies underscores a gap in the consideration of this demographic. None of the included studies simultaneously avoided both of the aforementioned issues, highlighting the critical need to enhance methodological and terminological standards. Sex-specific hormonal variations and relative weakness in neuromuscular strength primarily explain the performance disparities observed between elite female and male football players. Moreover, there are other variables that provide a better explanation for the differences between male and female football players than sex alone. Designing and conducting more personalized analyses for female football players should be a focus of future research.” |
| Limitations | 25 | p.14: the ***Limitations*** section of the manuscript. |
| Conclusions | 26 | p.15: the ***Conclusions*** section of the manuscript. |
| **Funding** | 27 | No funding |
