# Supplementary material for: Sex differences in association football: a scoping review
Source: PeerJ. 2025 Aug 27;13:e19976. doi: 10.7717/peerj.19976 (PMC12398286; doi:10.7717/peerj.19976)
Supplement: Supplemental Information 2 [file peerj-13-19976-s002.docx]

| **Table S1** Full search strategy for each database. | |
| --- | --- |
| Databases | Search strategy |
| Scopus | TITLE-ABS (((football* OR soccer*) AND ((male* AND female*) OR (man AND woman) OR (men AND women))) AND NOT (review OR rugby OR Gaelic OR patient* OR injury OR injuries OR pain OR fractures OR sprain* OR osteoarthritis* OR concussion* OR disease* OR disorder* OR dysfunction* OR epidemiology* OR prevalence*)) |
| PubMed | (((football*[Title/Abstract]) OR (soccer[Title/Abstract])) AND (((male*[Title/Abstract]) AND (female*[Title/Abstract])) OR ((man[Title/Abstract]) and (woman[Title/Abstract])) OR ((men[Title/Abstract]) AND (women[Title/Abstract])))) NOT ((review[Title/Abstract]) OR (rugby[Title/Abstract]) OR (Gaelic [Title/Abstract]) OR (patient*[Title/Abstract]) OR (injury[Title/Abstract]) OR (injuries[Title/Abstract]) OR (pain[Title/Abstract) OR (fractures[Title/Abstract]) OR (sprain*[Title/Abstract]) OR (osteoarthritis*[Title/Abstract]) OR (concussion*[Title/Abstract]) OR (disease*[Title/Abstract]) OR (disorder*[Title/Abstract]) OR (dysfunction*[Title/Abstract]) OR (epidemiology*[Title/Abstract]) OR (prevalence*[Title/Abstract])) |
| ScienceDirect | Title, abstract, keywords: ((football OR soccer) AND ((male AND female) OR (man and woman)) NOT (patient OR injury OR disease OR disorder) |
| Web of Science (Core Collection) | ((TI=(football*) OR AB=(football*) OR TI=(soccer) OR AB=(soccer)) AND (((TI=(male) OR AB=(male)) AND (TI=(female) OR AB=(female))) OR ((TI=(man) OR AB=(man)) AND (TI=(woman) OR AB=(woman))))) NOT ((TI=(review) OR AB=(review)) OR (TI=(rugby) OR AB=(rugby)) OR (TI=(Gaelic) OR AB=(Gaelic)) OR (TI=(patient) OR AB=(patient)) OR (TI=(injury) OR AB=(injury)) OR (TI=(pain) OR AB=(pain)) OR (TI=(fractures) OR AB=(fractures)) OR (TI=(sprain) OR AB=(sprain)) OR (TI=(osteoarthritis) OR AB=(osteoarthritis)) OR (TI=(concussion) OR AB=(concussion)) OR (TI=(disease) OR AB=(disease)) OR (TI=(disorder) OR AB=(disorder)) OR (TI=(dysfunction*) OR AB=(dysfunction*)) OR (TI=(epidemiology) OR AB=(epidemiology)) OR (TI=(prevalence) OR AB=(prevalence))) |
