# Supplementary material for: Sex differences in association football: a scoping review
Source: PeerJ. 2025 Aug 27;13:e19976. doi: 10.7717/peerj.19976 (PMC12398286; doi:10.7717/peerj.19976)
Supplement: Supplemental Information 3 [file peerj-13-19976-s003.docx]

| **Table S2** Summary of the included studies. | | | | | |
| --- | --- | --- | --- | --- | --- |
| Author (years) | Study design | Experimental protocol | Samples (age) | Main results | The use of sex and gender terminology |
| Mascherini et al. (2017) | Cross-sectional study | Anthropometrics parameters, skinfold thickness and bioimpedance | 18 F (26.2 ± 2.4)  18 M (26.9 ± 2.5) | F > M in fat mass index, vector length of lower limbs; F < M in fat-free mass index, body cell mass index, extracellular mass index, total body water index, and phase angle of lower limbs | Sex, gender, male, female |
| Baker et al. (2020) | Cross-sectional study | Anthropometric measures (height, body mass, tibiae lengths), dual energy x-ray absorptiometry (total body, lumbar spine, dual proximal femur) and peripheral quantitative computed tomography (4%, 38%, 66% dual tibiae) | 20 F (20.5 ± 1.5)  23 M (20.8 ± 2.3) | F < M in total body and hip areal peak bone mineral density, hip strength index and 4% and 38% tibia variables | Sex, male, female, man, woman |
| Hedt et al. (2022) | Cross-sectional study | Body composition and skeletal dimension analysis via dual-energy X-ray absorptiometry scans; balance and mobility were assessed using the Y-balance test and functional movement screen | 16 F (25 ± 3)  24 M (27 ± 5) | Differences between sexes were observed for all demographic variables with the exception of body mass index | Sex, male, female, man, woman |
| Schons et al. (2022) | Cross-sectional study | Anthropometric profile assessments and physical performance tests (e.g., jumping, linear sprint, change-of-direction and aerobic based test) | 44 F (23.9 ± 5.5)  48 M (22.8 ± 3.4) | Apart from the age and change-of-direction test, all other anthropometric and physical performance variables were significant different between sexes | Sex, gender, male, female, man, woman |
| Tornero-Aguilera et al. (2022) | Cross-sectional study | Six skinfold thickness measurement, single-frequency bioelectrical impedance analysis, and dual energy X-ray absorptiometry | 70 F (22.3 ± 3.2)  76 M (21.8 ± 5.0) | F > M in body fat mass | Sex, gender, male, female, man, woman |

**Table S2** (Continued)

| Author (years) | Study design | Experimental protocol | Samples (age) | Main results | The use of sex and gender terminology |
| --- | --- | --- | --- | --- | --- |
| Toro-Román et al. (2023a) | Cross-sectional study | Assessments of nutritional intake, anthropometry, body composition, and physical fitness (isometric strength, maximal aerobic capacity, and vertical jump) | 24 F (23.2 ± 4.1)  26 M (20.6 ± 2.7) | Anthropometry, body composition, and physical fitness differed between sexes; in males, there were significant correlations between body composition and aerobic capacity, while in females, there were correlations with isometric strength tests | Sex, gender, male, female, man, woman |
| Ahmad et al. (2024) | Cross-sectional study | To measure serum irisin levels and anthropometric metrics. | 12 F (26.2 ± 2.4)  18 M (26.2 ± 2.4) | F < M in fat mass; F = M in serum irisin concentrations | Sex, gender, male, female, man, woman |
| Petri et al. (2024a) | Cross-sectional study | Evaluated alterations in body composition during the season to determine the effects of a nutritional program led by a sport nutritionist | 44 F (26.2 ± 2.9)  44 M (27.0 ± 5.0) | F < M in fat mass; F > M in muscle mass | Gender, male, female, man, woman |
| Petri et al. (2024b) | Cross-sectional study | Stature, body mass, circumferences, skinfolds thicknesses, breadths, and somatotype were measured | 173 F (25.2 ± 5.1)  188 M (24.2 ± 4.8) | Females were balanced mesomorphs; males were ectomorphic mesomorphs | Sex, gender, male, female |
| Ichinose et al. (1998) | Cross-sectional study | The thickness and fibre pennation angle of the triceps brachii muscle and force output during elbow extensions were determined using a B-mode ultrasound apparatus and an isokinetic dynamometer, respectively | 20 F (23.7 ± 0.8)  16 M (20.8 ± 0.3) | F < M in maximum extension torque of elbow joint measured isokinetically at constant speeds of 60 °/s and 180 °/s | Sex, male, female, man, woman |
| Kanehisa et al. (2003) | Cross-sectional study | The thickness, fascicle angles of pennation, and fascicle length of the vastus lateralis and medial gastrocnemius muscles were determined from ulrasonograms | 20 F (23.7 ± 0.8)  18 M (20.8 ± 0.3) | F < M in the thickness and fascicle angles of the vastus lateralis and medial gastrocnemius muscles | Gender, male, female |

**Table S2** (Continued)

| Author (years) | Study design | Experimental protocol | Samples (age) | Main results | The use of sex and gender terminology |
| --- | --- | --- | --- | --- | --- |
| Hewett et al. (2006) | Cross-sectional study | Test dynamic hip control during the deceleration of three different types of singleleg landings | 19 F (20.0 ± 1.2)  17 M (21.4 ± 1.6) | F > M in hip adduction angles at initial contact during all three landings | Gender, male, female, man, woman |
| Hart et al. (2007) | Cross-sectional study | Collected surface electromyograms of the gluteus medius, vastus lateralis, lateral hamstring, and medial gastrocnemius during five trials of a 100 centimeter forward jump single leg landing | 8 F (22.0 ± 2.1)  8 M (19.1 ± 1.4) | F < M in average gluteus medius activity | Gender, male, female |
| Brophy et al. (2009) | Cross-sectional study | Evaluated hip range of motion, and hip and abdominal strength | 44 F (19.77 ± 1.29)  54 M (20.06 ± 1.58) | F >M in hip internal rotation | Gender, male, female |
| Zebis et al. (2011) | Cross-sectional study | Maximal voluntary static contraction for the hamstring and quadriceps in an isokinetic dynamometer, from which the maximal muscles strength and rate of force development were extracted | 11 F (22.0 ± 2.4)  12 M (24.5 ± 2.3) | F = M in maximal voluntary contraction hamstring/quadriceps; F < M in rate of force development hamstring/quadriceps | Gender, male, female, man, woman |
| Burfeind et al. (2012) | Cross-sectional study | Neuromuscular fitness was quantified through single leg balance assessment, time to generate peak torque, peak knee flexion and extension torque, hamstring to quadriceps ratio, and flexibility tests | 23 F (19.2 ± 1.2)  29 M (18.9 ± 1.1) | F > M in greater balance ability; F < M in peak knee extension and flexion torque; F = M in hamstring to quadriceps ratio and flexibility results | Gender, male, female, man, woman |
| Yilmaz et al. (2023) | Cross-sectional study | The knee strengths of both lower extremities were evaluated using isokinetic dynamometry at 60°/s and 180°/s | 26 F (23.35 ± 4.33)  26 M (21.85±4.16) | F = M in hamstring to quadriceps ratio and angle-specific ratio | Sex, gender, male, female, man, woman |

**Table S2** (Continued)

| Author (years) | Study design | Experimental protocol | Samples (age) | Main results | The use of sex and gender terminology |
| --- | --- | --- | --- | --- | --- |
| Makaraci et al. (2024) | Cross-sectional study | A functional movement screen test consisting of seven items and postural sway measures for dominant and non-dominant sides | 25 F (21.04 ± 1.24)  25 M (21.40 ± 1.94) | The scores of functional movement screen were positively correlated with postural stability in both sexes | Gender, male, female |
| Steding et al. (2010) | Cross-sectional study | Cardiac magnetic resonance and maximal incremental exercise test | 12 F (23 ± 4)  18 M (24 ± 5) | F < M in total heart volume, left ventricular mass, left and right ventricular end-diastolic volumes | Gender, male, female |
| Chamera et al. (2014) | Cross-sectional study | Evaluating metabolic response of football players during semi long distance outdoor running through biochemical liver characteristics | 8 F (21.9 ± 2.0)  8 M (18.4 ± 0.5) | Plasma levels of total and direct bilirubin were similar before and after the run regardless of the sex | Sex, gender, male, female, man, woman |
| Souglis et al. (2015) | Cross-sectional study | Blood samples were taken in the morning of the game day, immediately after the soccer game and 24 and 48 hours after the match | 21 F (22.9 ± 2.4)  22 M (23.1 ± 3.0) | F < M in peak tumor necrosis factor alpha; F = M in Interleukin 6, C-reactive protein and creatine kinase | Gender, male, female, man, woman |
| Chamera et al. (2015) | Cross-sectional study | Evaluate changes in the activity of: creatinine kinase, creatine kinase MB, lactate dehydrogenase, ahydroxybutyrate dehydrogenase cholinesterase and alkaline phosphatase in response to a semi-long distance outdoor run under aerobic conditions | 8 F (21.9 ± 2.0)  8 M (18.4 ± 0.5) | The creatine kinase-MB activity is statistically significantly increased in both F and M | Sex, gender, male, female, man, woman |
| Kostrzewa-Nowak et al. (2015) | Cross-sectional study | Determining C-reactive protein, total protein, and albumin plasma levels before and after 60-minute-long outdoor running | 8 F (21.9 ± 2.0)  8 M (18.4 ± 0.5) | Statistically significant increase in C-reactive protein level was found only in F | Sex, male, female, man, woman |

**Table S2** (Continued)

| Author (years) | Study design | Experimental protocol | Samples (age) | Main results | The use of sex and gender terminology |
| --- | --- | --- | --- | --- | --- |
| Dent et al. (2015) | Cross-sectional study | Participants performed four bouts of 6× 30-m maximal sprints spread equally over 40 min; heart rate, sprint time and sprint decrement were measured for each sprint and during the course of each bout; venous blood samples and muscle biopsies from the vastus lateralis were taken at rest, at 15 min and 2 h post-exercise | 8 F (20 ± 2)  7 M (19 ± 2) | Physiological responses such as heart rate and blood lactate were significantly elevated during and following the exercise but responded similarly between sexes. | Sex, male, female |
| Sanders et al. (2017) | Cross-sectional study | A graded maximal treadmill test measure VO2max and the repeated sprint test | 10 F (19.7 ± 0.9)  10 M (20.5 ± 0.7) | F < M in VO2max and average sprint time | Gender, male, female, man, woman |
| Souglis et al. (2018) | Cross-sectional study | Blood samples were taken before and after the match and daily for 5 days after the match | 30 F  30 M | F < M in all oxidative, inflammatory, and muscle damage indices | Sex, male, female, man, woman |
| Sansonio de Morais et al. (2018) | Cross-sectional study | Cardiac morphology was determined by echocardiography | 22 F (23.3 ± 4.5)  20 M (25.2 ± 3.9) | F < M in left ventricular intracavitary diameter and wall thickness | Gender, male, female |
| Mascherini et al. (2018) | Cross-sectional study | Body composition analysis and echocardiography | 25 F (26.2 ± 1.9)  25 M (26.5 ± 1.8) | F = M in systolic and diastolic parameters; F < M in all parameters regarding left ventricular dimensions | Sex, gender, male, female |
| Magal et al. (2020) | Cross-sectional study | Sprint interval cycling test was comprised of a warm-up, at 50 revolutions per min with no resistance, and six repeated 30-s wingate anaerobic tests separated by a 4-min recovery period between each sprint | 12 F (19.3 ± 1.0)  12 M (21.4 ± 2.4) | After defining want1 as the "baseline" measure, there were no sex differences in peak and mean power | Sex, male, female, man, woman |

**Table S2** (Continued)

| Author (years) | Study design | Experimental protocol | Samples (age) | Main results | The use of sex and gender terminology |
| --- | --- | --- | --- | --- | --- |
| Rodas et al. (2022) | Cross-sectional study | Targeted metabolic analysis of aminoacids, and tryptophan and phenylalanine metabolites | 28 F (25 ± 5)  23 M (25 ± 6) | There are sex differences in the tryptophan, cysteine and methionine metabolism, purine metabolism, and arginine and aminoacyl-trna biosynthesis | Sex, gender, male, female, man, woman |
| Mcfadden et al. (2024) | Cross-sectional study | Blood draws before preseason and every 4 weeks thereafter; workload was determined at all practices and games via heart rate and global positioning satellite monitoring systems | 26 F (19 ± 1)  23 M (20 ± 1) | F > M in total cortisol levels; F < M in ferritin levels | Sex, male, female, man, woman |
| Mujika et al. (2009) | Cross-sectional study | Tested for specific endurance (Yo-yoir1), sprint over 15 m, vertical jump without or with arm swing, agility (Agility-15m), and ball dribbling over 15 m | 17 F (23.1 ± 2.9)  17 M (23.8 ± 3.4) | F < M in Yo-yoir1 (m), Sprint-15m (m/s), Agility-15m (m/s), Ball-15m (m/s), vertical jump without arm swing (cm) and vertical jump with arm swing (cm) | Sex, gender, male, female, man, woman |
| Baumgart et al. (2014) | Cross-sectional study | Completed an incremental test to determine running velocities at 2 and 4 mmol/l blood lactate and maximum velocity as well as an interval shuttle run test to determine running distance | 14 F (21.4 ± 4.6)  13 M (22.8 ± 2.9) | F < M in running distance (m) of interval shuttle run test, maximum running velocity (km/h) and running velocity at 4 mmol/l blood lactate (km/h) of incremental test | Gender, male, female |
| Suchomel et al. (2015) | Cross-sectional study | Unloaded (< 1 kg) and loaded (20 kg) countermovement jumps | 22 F (18-23)  25 M (18-23) | F < M in jump height of unloaded and loaded countermovement jumps | Gender, male, female, man, woman |
| Mcfarland et al. (2016) | Cross-sectional study | Tested for 10 and 30 m sprint, T-test, pro-agility shuttle, counter movement jump and squat jump | 16 F (18-23)  20 M (18-23) | F < M in all tests’ performance | Gender, male, female |
| Suchomel et al. (2016) | Cross-sectional study | Squat jumps and countermovement jumps | 12 F (18-23)  21 M (18-23) | F < M in reactive strength index–modified of countermovement jumps | Sex, male, female, man, woman |

**Table S2** (Continued)

| Author (years) | Study design | Experimental protocol | Samples (age) | Main results | The use of sex and gender terminology |
| --- | --- | --- | --- | --- | --- |
| Condello et al. (2016) | Cross-sectional study | A 10-m sprint with a 60° change of direction at 5 m; vertical and mediolateral ground reaction force and contact time were measured | 12 F (21.0 ± 2.7)  12 M (19.6 ± 1.5) | F < M in mediolateral ground reaction force, performance cutting angle and minimum horizontal distance | Sex, gender, male, female, man, woman |
| Nagano et al. (2016) | Cross-sectional study | A shuttle run cutting task, and trunk accelerations (medio-lateral, vertical, and antero-posterior) were calculated | 8 F (18.9 ± 0.4)  8 M (21.4 ± 0.5) | F > M in peak vertical acceleration | Gender, male, female |
| Cardoso de Araujo et al. (2018) | Cross-sectional study | Tested for linear and nonlinear sprint, squat and countermovement jump, core endurance, as well as incremental and intermittent endurance capacities | 29 F  47 M | F < M in all sprints and jumps performance, and most indicators of core, incremental and intermittent endurance | Sex, male, female, man, woman |
| Baumgart et al. (2018) | Cross-sectional study | A 30 m linear sprint; from timing gate derived sprint times, force-velocity and power-velocity relationships, as well as theoretical maximum running velocity, force, and power data were computed by an inverse dynamic approach applied to the center of mass | 14 F (23.2 ± 4.2)  13 M (25.7 ± 4.7) | F < M in sprint times, theoretical maximum running velocity, horizontal force and mechanical power | Gender, male, female |
| Cardoso de Araujo et al. (2019) | Cross-sectional study | The interval shuttle run test with monitored heart rate | 34 F  33 M | F < M in the interval shuttle run test performance; F > M in submaximal heart rate | Sex, male, female, man, woman |
| Devismes et al. (2019) | Cross-sectional study | Two maximal 60-m sprints; the theoretical maximal force and velocity, maximal power, maximal ratio of force and the slope of the force-velocity profile were computed | 63 F (20.8 ± 4.5)  100 M (24.6 ± 4.4) | F > M in maximal velocity and maximal power | Sex, male, female, man, woman |

**Table S2** (Continued)

| Author (years) | Study design | Experimental protocol | Samples (age) | Main results | The use of sex and gender terminology |
| --- | --- | --- | --- | --- | --- |
| Dolci et al. (2021) | Cross-sectional study | Movement economy as extrapolated from oxygen uptake during in-line running and running with changes of directions at 8.4km/h mean speed | 16 F (18.9 ± 2.1)  14 M (18.1 ± 1.1) | F > M in movement economy during 10 m shuttle runs | Sex, male, female |
| Putukian et al. (2000) | Cross-sectional study | Neuropsychological tests symptom checklist compared at baseline with those after the practice sessions | 56 F  44 M | There are no acute effects of heading on cognitive function for both sexes | Sex, gender, male, female, man, woman |
| Barfield et al. (2002) | Cross-sectional study | Participants took a two-step angled approach of 45-60 degrees to a stationary soccer ball positioned between two force platforms and kicked the ball with the instep portion of the foot as hard as possible into netting which was draped from the ceiling | 6 F (19-22)  2 M (19-22) | F < M in maximum toe velocity, ball contact ball velocity, mean toe velocity, mean toe acceleration, and ankle velocity at ball contact | Gender, male, female, man, woman |
| Orloff et al. (2008) | Cross-sectional study | Participants were filmed in both the sagittal and posterior views while performing a maximal instep kick | 12 F  11 M | F = M in plant leg position; F > M in trunk lean, plant leg angle, and medial-lateral ground reaction force | Sex, male, female, man, woman |
| Gheidi et al. (2010) | Cross-sectional study | Compare selected kinematic parameters of the kicking foot during the performance of successful and unsuccessful penalty kick from a 6 m distance | 7 F (23 ± 2.4)  7 M (23 ± 1.7) | Accurate kicks had lower peak knee velocities than unsuccessful kicks for both sexes | Sex, gender, male, female, man, woman |
| Sakamoto et al. (2013) | Cross-sectional study | Compared the technical characteristics of ball impact during instep and inside kicks | 17 F (19–25)  17 M (19–25) | F < M in ball velocity, foot velocity immediately before impact, striking mass, and average ball-to-foot velocity ratio | Gender, male, female |

**Table S2** (Continued)

| Author (years) | Study design | Experimental protocol | Samples (age) | Main results | The use of sex and gender terminology |
| --- | --- | --- | --- | --- | --- |
| Bretzin et al. (2016) | Cross-sectional study | Participants assessed for head-neck anthropometric and neck strength measurements in 6 directions; headed the ball 10 times at 2 ball speeds while wearing an accelerometer secured to their head | 8 F (20.25 ± 0.70)  5 M (19.20 ± 1.09) | F < M in isometric head-neck segment strength | Sex, gender, male, female, man, woman |
| Reynolds et al. (2017) | Cross-sectional study | Participants wore mastoid patch accelerometers that measured head impacts during team events | 7 F (20.43 ± 1.27)  12 M (20.33 ± 1.23) | F = M in the frequency or severity of head impacts during training | Gender, male, female, man, woman |
| Saunders et al. (2020) | Cross-sectional study | Sensors collected linear and rotational accelerations and frequency of head impacts | 16 F (19.94 ± 1.06)  12 M (20.25 ± 1.14) | F < M in the frequency of heading during matches | Sex, male, female, man, woman |
| Nelson et al. (2020) | Cross-sectional study | Determine the magnitude and frequency of head impacts based on player position and type of play (offense, defense, transition) | 16 F (19.94 ± 1.06)  12 M (20.25 ± 1.14) | Defenders in both sexes performing the most headers | Sex, male, female, man, woman |
| Langdon et al. (2022) | Cross-sectional study | Video analysis, combined with a structured electronic registration tool and observation training, was used to comprehensively document heading characteristics in official matches of Dutch national teams | 20 F matches  20 M matches | F < M in maximum heading exposure | Sex, male, female |
| Navandar et al. (2022) | Cross-sectional study | Participants performed maximal effort instep kicks while motion capture and post-impact ball velocities data were recorded | 23 F (22 ± 5)  19 M (21 ± 2) | F = M in hip and knee joint moments | Sex, male, female |
| Iitake et al. (2022) | Cross-sectional study | Joint moments of the kicking leg were computed and normalized by the body mass and height | 7 F (20.0 ± 0.0)  7 M (20.1 ± 0.4) | F < M in resultant ball velocity, run-up velocity and foot–ball velocity ratio | Sex, male, female |

**Table S2** (Continued)

| Author (years) | Study design | Experimental protocol | Samples (age) | Main results | The use of sex and gender terminology |
| --- | --- | --- | --- | --- | --- |
| Jackson et al. (2023) | Cross-sectional study | Xpatch sensors applied over the participants’ right mastoid processes for training and matches provided the frequency and biomechanics of all of the head impacts over 10 g | 16 F (19 ± 1.05)  14 M (20 ± 1.07) | F < M in the frequency of headers | Sex, male, female, man, woman |
| Peek et al. (2024) | Cross-sectional study | Video analysis of all observed headers, attempted headers and other head impacts during eight FIFA World Cup matches where the same national teams competed | 4 F matches  4 M matches | F < M in controlled headers | Sex, man, woman |
| Siegle et al. (2012) | Cross-sectional study | For each stoppage, duration, type (e.g. Free kick, goal kick, etc.), mode (knock out vs. Round robin), sex, and location were registered | 16 F matches  32 M matches | F > M in the stoppage incidence | Sex, male, female |
| Bradley et al. (2014) | Cross-sectional study | Players were tracked during UEFA Champions League matches using a multi-camera system | 59 F  54 M | F < M in total running distance | Gender, male, female |
| Jastrzębski et al. (2017) | Cross-sectional study | Reported sex differences the distances covered in the respective speed zones during small-sided games using default and individualized time–motion analyses | 8 F (19.1 ± 3.14)  8 M (27.5 ± 5.14) | F < M in the mean running velocity at lactate threshold | Sex, gender, male, female, man, woman |
| Mcfadden et al. (2020) | Cross-sectional study | Evaluated by the Polar teampro system, utilizing Global Positioning Satellite technology and heart rate monitoring to determine training loads | 16 F (19.3 ± 1.4)  12 M (20.1 ± 1.3) | F = M in total running distance | Sex, male, female, man, woman |

**Table S2** (Continued)

| Author (years) | Study design | Experimental protocol | Samples (age) | Main results | The use of sex and gender terminology |
| --- | --- | --- | --- | --- | --- |
| Pappalardo et al. (2021) | Cross-sectional study | Analyzed the spatio-temporal events during matches in the World Cups to compare male and female teams based on their technical performance | 44 F matches  64 M matches | F > M in the stoppage incidence; F < M in the number of fouls | Sex, gender, male, female, man, woman |
| Coulomb-Cabagno et al. (2006) | Cross-sectional study | Examining observed aggression in team sports | 15 F matches  15 M matches | F < M in aggressive behaviors | Sex, gender, male, female, man, woman |
| Adegbesan (2007) | Cross-sectional study | Identify sources of self-confidence within the sport-confidence framework of Vealey | 99 F (21.61 ± 2.21)  103 M (22.63 ± 2.91) | F > M in all sources of sport confidence factors except situational favourableness | Gender, male, female |
| López-Gajardo et al. (2021) | Cross-sectional study | To assess perceived justice, an adapted and translated Spanish version of Colquitt’s Justice Questionnaire was used; to assess satisfaction with the coach, the adapted Spanish version of the scale was used | 211 F (23.81 ± 4.53)  227 M (25.27 ± 4.52) | F grant more importance to relational and motivational aspects | Gender, male, female, man, woman |
| De la Vega et al. (2022) | Cross-sectional study | Examine differences in achievement motivation (measured with the Objective Achievement Motivation Test, OLMT, Schuhfried) and competitiveness between sexes | 11 F (22.6 ± 5.20)  27 M (28.4 ± 4.60) | F < M in the motivation through competition and in self-reported competitiveness | Gender, male, female, man, woman |
| Avugos et al. (2022) | Cross-sectional study | Recorded the goalkeeper’s dive direction during penalties and accurately measured the location of the ball as it crossed the goal line | 131 F  435 M | F > M in tendency of kicking to the goalkeepers’ right | Sex, gender, male, female, man, woman |

**Table S2** (Continued)

| Author (years) | Study design | Experimental protocol | Samples (age) | Main results | The use of sex and gender terminology |
| --- | --- | --- | --- | --- | --- |
| Jin et al. (2023) | Cross-sectional study | To assess visual attention, a multiple object tracking task was used with four targets among a total of 10 objects moving at a fixed speed of 10◦/s in random directions across a computer monitor screen | 32 F (21.86 ± 2.47)  32 M (22.35 ± 2.31) | F = M in tracking performance | Sex, male, female, man, woman |
| Bonet et al. (2024) | Cross-sectional study | International Neuropsychiatric Interview 5.0, Hamilton Anxiety Scale, Hamilton Depression Scale, Barrat’s Impulsivity Scale and Sensitivity to Punishment and Sensitivity to Reward Questionnaire were used, to assess the state of mental health. | 21 F (23.0 ± 5.2)  21 M (23.5 ± 3.8) | F > M in the somatic anxiety | Sex, male, female, man, woman |
| Ros et al. (2013) | Cross-sectional study | Assess the responsiveness of the 1-leg hop test and the square hop test to fatiguing intermittent aerobic work and during the recovery period | 10 F (21.8 ± 4.8)  10 M (20.7 ± 3.4) | F= M in the trends of jumping performance | Sex, gender, male, female, man, woman |
| Koikawa et al. (2016) | Cross-sectional study | The Pittsburgh Sleep Quality Index was used to assess subjective sleep quality, the Japanese Version of the Epworth Sleepiness Scale was used to evaluate subjective sleepiness, and Health-related quality-of-life was evaluated through the short form-8 | 30 F (19.8 ± 1.2)  64 M (19.8 ± 1.2) | F > M in subjective sleepiness; F < M in sleep quality | Sex, gender, male, female |
| Gomez-Hixson et al. (2020) | Cross-sectional study | Actual dietary intake was determined by the analysis of a 3-day food record | 31 F (18-22)  47 M (18-22) | F > M in intakes of added sugars, saturated fats, and vitamin C; F < M in intakes of potassium, sodium, iron, magnesium, vitamin D | Sex, gender, male, female |

**Table S2** (Continued)

| Author (years) | Study design | Experimental protocol | Samples (age) | Main results | The use of sex and gender terminology |
| --- | --- | --- | --- | --- | --- |
| Biggins et al. (2022) | Cross-sectional study | Daily monitoring via actigraphy and subjective sleep and well-being measures were obtained for 1 week in Ireland (baseline), and for the duration of an international soccer tournament | 20 F (22.2 ± 2.0)  21 M (20.8 ± 1.3) | F > M in presleep tension–anxiety | Sex, male, female, man, woman |
| Toro-Román et al. (2022) | Cross-sectional study | Mo and Zn concentrations were determined by inductively coupled plasma mass spectrometry; erythrocytes, platelets, creatine kinase, and lactate dehydrogenase values were determined by automatic cell counter and spectrophotometric techniques | 70 F (23.37 ± 3.95)  68 M (20.61 ± 2.66) | F > M in concentrations of zinc in erythrocytes; F < M in concentrations of molybdenum and zinc in plasma and urine | Sex, gender, male, female, man, woman |
| Kwon et al. (2023) | Cross-sectional study | Players were instructed to complete 3-day food diaries, which were subsequently analyzed and compared to UEFA expert group-issued nutrition guidelines for soccer players | 23 F (19.4 ± 1.5)  13 M (19.7 ± 1.3) | F < M in the daily total protein intake | Sex, male, female, man, woman |
| Robles-Gil et al. (2023) | Cross-sectional study | Three assessments were carried out during the season (beginning, middle and end); Al concentrations in plasma, urine, erythrocytes and platelets were determined | 24 F (23.37 ± 3.95)  22 M (20.61 ± 2.66) | F < M in intake and plasma concentrations of aluminum | Sex, gender, male, female, man, woman |
| Toro-Román et al. (2023b) | Cross-sectional study | Anthropometry, body composition, nutritional intake, physical condition, female hormones and hematology were evaluated, as well as Cu determination | 24 F (23.21 ± 4.11)  22 M (20.62 ± 2.66) | F < M in amount of copper in urine | Sex, male, female, man, woman |

**Table S2** (Continued)

| Author (years) | Study design | Experimental protocol | Samples (age) | Main results | The use of sex and gender terminology |
| --- | --- | --- | --- | --- | --- |
| Toro-Román et al. (2023c) | Cross-sectional study | Erythrocyte, haemoglobin, platelet, plateletcrit, ferritin and serum iron values were determined; cadmium and lead concentrations were quantified by inductively coupled plasma mass spectrometry | 70 F (23.37 ± 3.95)  68 M (20.61 ± 2.66) | F > M in concentrations of cadmium and lead in plasma, erythrocytes, and platelets; F < M in hemoglobin, erythrocytes, ferritin, and serum iron | Sex, gender, male, female, man, woman |
| Sebastia-Rico et al. (2024) | Cross-sectional study | Measure and fluid balance and urine values during training at high and low temperatures | 29 F  25 M | F < M in fluid intake, sweating rates, and weight loss | Sex, gender, male, female, man, woman |
| Toro-Román et al. (2024) | Cross-sectional study | Extracellular (plasma and urine) and intracellular (erythrocytes and platelets) Zn concentrations were determined, as well as physical fitness and several blood parameters | 24 F (23.37 ± 3.95)  22 M (20.61 ± 2.66) | F > M in concentrations of zinc in erythrocyte; F < M in concentrations of zinc in plasma and urine | Sex, male, female, man, woman |
| **Abbreviations:** F, females; M, males; <, significantly lower than; >, significantly greater than; =, no significant difference. | | | | | |

**References**

Adegbesan, O. A. (2007). Sources of sport confidence of elite male and female soccer players in Nigeria. *Eur J Sci Res, 18*(2), 217-222.

Ahmad, M. N. and Abu Al Haija, D. M. (2024). Circulating irisin and its connection with indices of body composition in aerobic and anaerobic endurance professional athletes: a case-control study. *Health, sport, rehabilitation, 10*(1), 27-38. doi:10.58962/hsr.2024.10.1.27-38

Avugos, S., Azar, O. H., Sher, E., Gavish, N. and Bar-Eli, M. (2022). Detecting patterns in the behaviour of goalkeepers and kickers in the penalty shootout: a between-gender comparison among score situations. *Int J Sport Exerc Psychol, 21*(2), 196-216. doi:10.1080/1612197x.2022.2066704

Baker, B. S., Chen, Z., Larson, R. D., Bemben, M. G. and Bemben, D. A. (2020). Sex differences in bone density, geometry, and bone strength of competitive soccer players. *J Musculoskelet Neuronal Interact, 20*(1), 62-76.

Barfield, W. R., Kirkendall, D. T. and Yu, B. (2002). Kinematic instep kicking differences between elite female and male soccer players. *J Sport Sci Med, 1*(3), 72-79. doi:10.1097/00005768-200105001-00576

Baumgart, C., Freiwald, J. and Hoppe, M. W. (2018). Sprint mechanical properties of female and different aged male top-level german soccer players. *Sports, 6*(4). doi:10.3390/sports6040161

Baumgart, C., Hoppe, M. W. and Freiwald, J. (2014). Different endurance characteristics of female and male german soccer players. *Biol Sport, 31*(3), 227-232. doi:10.5604/20831862.1111851

Biggins, M., Purtill, H., Fowler, P., O'Sullivan, K. and Cahalan, R. (2022). Impact of long-haul travel to international competition on sleep and recovery in elite male and female soccer athletes. *Int J Sports Physiol Perform, 17*(9), 1361-1370. doi:10.1123/ijspp.2021-0165

Bonet, L., Benito, A., Uso, H., Peraire, M., Haro, G. and Almodovar-Fernandez, I. (2024). Mental health in first- and second-division soccer players: a cross-sectional study. *Sports, 12*(4). doi:10.3390/sports12040106

Bradley, P. S., Dellal, A., Mohr, M., Castellano, J. and Wilkie, A. (2014). Gender differences in match performance characteristics of soccer players competing in the UEFA Champions League. *Hum. Mov. Sci., 33*, 159-171. doi:10.1016/j.humov.2013.07.024

Bretzin, A. C., Mansell, J. L., Tierney, R. T. and McDevitt, J. K. (2016). Sex differences in anthropometrics and heading kinematics among Division I soccer athletes. *Sports Health, 9*(2), 168-173. doi:10.1177/1941738116678615

Brophy, R. H., Chiaia, T. A., Maschi, R., Dodson, C. C., Oh, L. S., Lyman, S., Allen, A. A. and Williams, R. J. (2009). The core and hip in soccer athletes compared by gender. *Int J Sports Med, 30*(9), 663-667. doi:10.1055/s-0029-1225328

Burfeind, K., Hong, J. G. and Stavrianeas, S. (2012). Gender differences in the neuromuscular fitness profiles of NCAA Division III soccer players. *Isokinet Exerc Sci, 20*(2), 115-120. doi:10.3233/ies-2012-0449

Cardoso de Araujo, M., Baumgart, C., Freiwald, J. and Hoppe, M. W. (2019). Contrasts in intermittent endurance performance and heart rate response between female and male soccer players of different playing levels. *Biol Sport, 36*(4), 323-331. doi:10.5114/biolsport.2019.88755

Cardoso de Araujo, M., Baumgart, C., Jansen, C. T., Freiwald, J. and Hoppe, M. W. (2018). Sex differences in physical capacities of German Bundesliga soccer players. *J Strength Cond Res, 34*(8), 2329-2337. doi:10.1519/JSC.0000000000002662

Chamera, T., Spieszny, M., Klocek, T., Kostrzewa-Nowak, D., Nowak, R., Lachowicz, M., Buryta, R. and Cięszczyk, P. (2014). Could biochemical liver profile help to assess metabolic response to aerobic effort in athletes? *J Strength Cond Res, 28*(8), 2180-2186. doi:10.1519/jsc.0000000000000398

Chamera, T., Spieszny, M., Klocek, T., Kostrzewa-Nowak, D., Nowak, R., Lachowicz, M., Buryta, R., Ficek, K., Eider, J., Moska, W. and Cięszczyk, P. (2015). Post-effort changes in activity of traditional diagnostic enzymatic markers in football players' blood. *J Med Biochem, 34*(2), 179-190. doi:10.2478/jomb-2014-0035

Condello, G., Kernozek, T. W., Tessitore, A. and Foster, C. (2016). Biomechanical analysis of a change-of-direction task in collegiate soccer players. *Int J Sports Physiol Perform, 11*(1), 96-101. doi:10.1123/ijspp.2014-0458

Coulomb‐Cabagno, G. and Rascle, O. (2006). Team sports players' observed aggression as a function of gender, competitive level, and sport type. *Journal of Applied Social Psychology, 36*(8), 1980-2000. doi:10.1111/j.0021-9029.2006.00090.x

De la Vega, R., Gómez, J., Vaquero-Cristobal, R., Horcajo, J. and Abenza-Cano, L. (2022). Objective comparison of achievement motivation and competitiveness among semi-professional male and female football players. *Sustainability, 14*(9). doi:10.3390/su14095258

Dent, J. R., Edge, J. A., Hawke, E., McMahon, C. and Mündel, T. (2015). Sex differences in acute translational repressor 4E-BP1 activity and sprint performance in response to repeated-sprint exercise in team sport athletes. *J Sci Med Sport, 18*(6), 730-736. doi:10.1016/j.jsams.2014.10.006

Devismes, M., Aeles, J., Philips, J. and Vanwanseele, B. (2019). Sprint force-velocity profiles in soccer players: impact of sex and playing level. *Sports Biomech, 20*(8), 947-957. doi:10.1080/14763141.2019.1618900

Dolci, F., Kilding, A., Spiteri, T., Chivers, P., Piggott, B., Maiorana, A. and Hart, N. H. (2021). Characterising running economy and change of direction economy between soccer players of different playing positions, levels and sex. *Eur J Sport Sci, 22*(8), 1167-1176. doi:10.1080/17461391.2021.1953151

Gomez-Hixson, K., Biagioni, E. and Brown, M. L. (2020). Significant differences in dietary intake of NCAA Division III soccer players compared to recommended levels. *J Am Coll Health, 70*(1), 150-157. doi:10.1080/07448481.2020.1728279

Hart, J. M., Garrison, J. C., Kerrigan, D. C., Palmieri-Smith, R. and Ingersoll, C. D. (2007). Gender differences in gluteus medius muscle activity exist in soccer players performing a forward jump. *Res Sports Med, 15*(2), 147-155. doi:10.1080/15438620701405289

Hedt, C. A., Le, J. T., Heimdal, T., Vickery, J., Orozco, E., McCulloch, P. C. and Lambert, B. S. (2022). Sex-related anthropometrics in a lower-body mobility assessment among professional soccer athletes. *Int J Sports Phys Ther, 17*(3), 474-482. doi:10.26603/001c.32595

Hewett, T. E., Ford, K. R., Myer, G. D., Wanstrath, K. and Scheper, M. (2006). Gender differences in hip adduction motion and torque during a single-leg agility maneuver. *J Orthop Res, 24*(3), 416-421. doi:10.1002/jor.20056

Ichinose, Y., Kanehisa, H., Ito, M., Kawakami, Y. and Fukunaga, T. (1998). Morphological and functional differences in the elbow extensor muscle between highly trained male and female athletes. *Eur J Appl Physiol Occup Physiol, 78*(2), 109-114. doi:10.1007/s004210050394

Iitake, T., Hioki, M., Takahashi, H. and Nunome, H. (2022). Sex difference in soccer instep kicking. *J Sports Sci, 40*(20), 2217-2224. doi:10.1080/02640414.2022.2139881

Jackson, B. C., Rogerson, C. E., Bradney, D. A., Breedlove, K. M. and Bowman, T. G. (2023). Preparedness during head impacts in intercollegiate men’s and women’s soccer athletes. *Biomechanics, 3*(1), 45-51. doi:10.3390/biomechanics3010004

Jastrzębski, Z. and Radzimiński, Ł. (2017). Default and individual comparison of physiological responses and time-motion analysis in male and female soccer players during small-sided games. *J Hum Sport Exerc, 12*(4), 1176-1185. doi:10.14198/jhse.2017.124.04

Jin, P., Ji, Z., Wang, T. and Zhu, X. (2023). Association between sports expertise and visual attention in male and female soccer players. *PeerJ, 11*, e16286. doi:10.7717/peerj.16286

Kanehisa, H., Muraoka, Y., Kawakami, Y. and Fukunaga, T. (2003). Fascicle arrangements of vastus lateralis and gastrocnemius muscles in highly trained soccer players and swimmers of both genders. *Int J Sports Med, 24*(2), 90-95. doi:10.1055/s-2003-38197

Koikawa, N., Shimada, S., Suda, S., Murata, A. and Kasai, T. (2016). Sex differences in subjective sleep quality, sleepiness, and health-related quality of life among collegiate soccer players. *Sleep Biol Rhythms, 14*(4), 377-386. doi:10.1007/s41105-016-0068-4

Kostrzewa-Nowak, D., Nowak, R., Chamera, T., Buryta, R., Moska, W. and Cięszczyk, P. (2015). Post-effort chances in C-reactive protein level among soccer players at the end of the training season. *J Strength Cond Res, 29*(5), 1399-1405. doi:10.1519/jsc.0000000000000753

Kwon, J., Nishisaka, M. M., McGrath, A. F., Kristo, A. S., Sikalidis, A. K. and Reaves, S. K. (2023). Protein intake in NCAA Division 1 soccer players: assessment of daily amounts, distribution patterns, and leucine levels as a quality indicator. *Sports, 11*(2). doi:10.3390/sports11020045

Langdon, S., Goedhart, E., Oosterlaan, J. and Konigs, M. (2022). Heading exposure in elite football (soccer): a study in adolescent, young adult, and adult male and female players. *Med Sci Sports Exerc, 54*(9), 1459-1465. doi:10.1249/MSS.0000000000002945

López-Gajardo, M. Á., Ponce-Bordón, J. C., Rubio-Morales, A., Llanos-Muñoz, R. and Díaz-García, J. (2021). The role of perceived justice on satisfaction with the coach: gender differences in a longitudinal study. *Sustainability, 14*(1). doi:10.3390/su14010401

Magal, M., Liette, N. C., Crowley, S. K., Hoffman, J. R. and Thomas, K. S. (2020). Sex-based performance responses to an acute sprint interval cycling training session in collegiate athletes. *Res Q Exerc Sport, 92*(3), 469-476. doi:10.1080/02701367.2020.1751026

Makaraci, Y., Nas, K., GÜNdÜZ, K. and İLeri, M. (2024). Relationship between functional movement screen scores and postural stability in football players: an asymmetrical approach. *Balt J Health Phys Act, 16*(1), Article6-Article6. doi:10.29359/bjhpa.16.1.06

Mascherini, G., Castizo-Olier, J., Irurtia, A., Petri, C. and Galanti, G. (2017). Differences between the sexes in athletes' body composition and lower limb bioimpedance values. *Muscles, Ligaments Tendons J, 7*(4), 573-581. doi:10.11138/mltj/2017.7.4.573

Mascherini, G., Petri, C. and Galanti, G. (2018). Link between body cellular mass and left ventricular hypertrophy in female and male athletes. *J Sports Med Phys Fitness, 59*(1), 164-170. doi:10.23736/S0022-4707.18.08259-2

McFadden, B. A., Walker, A. J., Bozzini, B. N., Sanders, D. J. and Arent, S. M. (2020). Comparison of internal and external training loads in male and female collegiate soccer players during practices vs. games. *J Strength Cond Res, 34*(4), 969-974. doi:10.1519/JSC.0000000000003485

McFadden, B. A., Walker, A. J., Cintineo, H. P., Bozzini, B. N., Sanders, D. J., Chandler, A. J. and Arent, S. M. (2024). Sex differences in physiological responses to a national collegiate athletic association Division I soccer season. *J Strength Cond Res*. doi:10.1519/JSC.0000000000004882

McFarland, I. T., Dawes, J. J., Elder, C. L. and Lockie, R. G. (2016). Relationship of two vertical jumping tests to sprint and change of direction speed among male and female collegiate soccer players. *Sports, 4*(1). doi:10.3390/sports4010011

Mujika, I., Santisteban, J., Impellizzeri, F. M. and Castagna, C. (2009). Fitness determinants of success in men's and women's football. *J Sports Sci, 27*(2), 107-114. doi:10.1080/02640410802428071

Gheidi, N. and Sadeghi, H. (2010). Kinematic comparison of successful and unsuccessful instep kick in indoor soccer. *Am J Applied Sci, 7*(10), 1334-1340. doi:10.3844/ajassp.2010.1334.1340

Nagano, Y., Sasaki, S., Higashihara, A. and Ishii, H. (2016). Gender differences in trunk acceleration and related posture during shuttle run cutting. *Int Biomech, 3*(1), 33-39. doi:10.1080/23335432.2016.1191372

Navandar, A., Kipp, K. and Navarro, E. (2022). Hip and knee joint angle patterns and kicking velocity in female and male professional soccer players: a principal component analysis of waveforms approach. *J Sports Sci, 40*(17), 1919-1930. doi:10.1080/02640414.2022.2121022

Nelson, K. M., Daidone, E. H. K., Breedlove, K. M., Bradney, D. A. and Bowman, T. G. (2020). Head impact characteristics based on player position in collegiate soccer athletes. *Int J Athl Ther Trai, 26*(2), 111-115. doi:10.1123/ijatt.2019-0095

Orloff, H., Sumida, B., Chow, J., Habibi, L., Fujino, A. and Kramer, B. (2008). Ground reaction forces and kinematics of plant leg position during instep kicking in male and female collegiate soccer players. *Sports Biomech, 7*(2), 238-247. doi:10.1080/14763140701841704

Pappalardo, L., Rossi, A., Natilli, M. and Cintia, P. (2021). Explaining the difference between men's and women's football. *PLoS one, 16*(8), e0255407. doi:10.1371/journal.pone.0255407

Peek, K., Georgieva, J., Serner, A. and Orest, F. (2024). Differences in the technical performance of heading between men and women football players during FIFA World Cup 2022 and FIFA Women's World Cup 2023 matches. *BMJ Open Sport Exerc Med, 10*(3), e002066. doi:10.1136/bmjsem-2024-002066

Petri, C., Campa, F., Holway, F., Pengue, L. and Arrones, L. S. (2024a). ISAK-based anthropometric standards for elite male and female soccer players. *Sports, 12*(3). doi:10.3390/sports12030069

Petri, C., Pengue, L., Bartolini, A., Pistolesi, D. and Arrones, L. S. (2024b). Body composition changes in male and female elite soccer players: effects of a nutritional program led by a sport nutritionist. *Nutrients, 16*(3). doi:10.3390/nu16030334

Putukian, M., Echemendia, R. J. and Mackin, S. (2000). The acute neuropsychological effects of heading in soccer: a pilot study. *Clin J Sport Med, 10*(2), 104-109. doi:10.1097/00042752-200004000-00004

Reynolds, B. B., Patrie, J., Henry, E. J., Goodkin, H. P., Broshek, D. K., Wintermark, M. and Druzgal, T. J. (2017). Effects of sex and event type on head impact in collegiate soccer. *Orthop J Sports Med, 5*(4), 2325967117701708. doi:10.1177/2325967117701708

Robles-Gil, M. C., Toro-Roman, V., Maynar-Marino, M., Siquier-Coll, J., Bartolome, I. and Grijota, F. J. (2023). Aluminum concentrations in male and female football players during the season. *Toxics, 11*(11). doi:10.3390/toxics11110920

Rodas, G., Ferrer, E., Reche, X., Sanjuan-Herraez, J. D., McCall, A. and Quintas, G. (2022). A targeted metabolic analysis of football players and its association to player load: comparison between women and men profiles. *Front Physiol, 13*, 923608. doi:10.3389/fphys.2022.923608

Ros, A. G., Holm, S. E., Fridén, C. and Heijne, A. I. (2013). Responsiveness of the one-leg hop test and the square hop test to fatiguing intermittent aerobic work and subsequent recovery. *J Strength Cond Res, 27*(4), 988-994. doi:10.1519/JSC.0b013e31825feb5b

Sakamoto, K. and Asai, T. (2013). Comparison of kicking motion characteristics at ball impact between female and male soccer players. *Int J Sports Sci Coa, 8*(1), 63-76. doi:10.1260/1747-9541.8.1.63

Sanders, G. J., Turner, Z., Boos, B., Peacock, C. A., Peveler, W. and Lipping, A. (2017). Aerobic capacity is related to repeated sprint ability with sprint distances less than 40 meters. *Int J Exerc Sci, 10*(2), 197-204. doi: 10.70252/gcdn2088

Sansonio de Morais, A., Ferreira, G. A., Lima-Silva, A. E. and Gomes Filho, A. (2018). Gender-related cardiac dimension differences between female and male professional soccer players. *J Sports Med Phys Fitness, 58*(9), 1354-1359. doi:10.23736/S0022-4707.17.07422-9

Saunders, T. D., Le, R. K., Breedlove, K. M., Bradney, D. A. and Bowman, T. G. (2020). Sex differences in mechanisms of head impacts in collegiate soccer athletes. *Clin Biomech, 74*, 14-20. doi:10.1016/j.clinbiomech.2020.02.003

Schons, P., Birk Preissler, A. A., Oliveira, R., Brito, J. P., Clemente, F. M., Droescher de Vargas, G., Moraes Klein, L. and Kruel, L. F. M. (2022). Comparisons and correlations between the anthropometric profile and physical performance of professional female and male soccer players: Individualities that should be considered in training. *Int J Sports Sci Coa, 18*(6), 2004-2014. doi:10.1177/17479541221131649

Sebastia-Rico, J., Soriano, J. M., Sanchis-Chorda, J., Garcia-Fernandez, A. F., Lopez-Mateu, P., de la Cruz Marcos, S. and Martinez-Sanz, J. M. (2024). Analysis of fluid balance and urine values in elite soccer players: impact of different environments, playing positions, sexes, and competitive levels. *Nutrients, 16*(6). doi:10.3390/nu16060903

Siegle, M. and Lames, M. (2012). Influences on frequency and duration of game stoppages during soccer. *Int J Perform Anal Spor, 12*(1), 101-111. doi:10.1080/24748668.2012.11868586

Souglis, A., Bogdanis, G. C., Chryssanthopoulos, C., Apostolidis, N. and Geladas, N. D. (2018). Time course of oxidative stress, inflammation, and muscle damage markers for 5 days after a soccer match: effects of sex and playing position. *J Strength Cond Res, 32*(7), 2045-2054. doi:10.1519/JSC.0000000000002436

Souglis, A., Papapanagiotou, A., Bogdanis, G. C., Travlos, A. K., Apostolidis, N. G. and Geladas, N. D. (2015). Comparison of inflammatory responses to a soccer match between elite male and female players. *J Strength Cond Res, 29*(5), 1227-1233. doi:10.1519/jsc.0000000000000767

Steding, K., Engblom, H., Buhre, T., Carlsson, M., Mosén, H., Wohlfart, B. and Arheden, H. (2010). Relation between cardiac dimensions and peak oxygen uptake. *J Cardiovasc Magn Reson, 12*(1), 8. doi:10.1186/1532-429X-12-8

Suchomel, T. J., Sole, C. J., Bailey, C. A., Grazer, J. L. and Beckham, G. K. (2015). A comparison of reactive strength index-modified between six U.S. collegiate athletic teams. *J Strength Cond Res, 29*(5), 1310-1316. doi:10.1519/jsc.0000000000000761

Suchomel, T. J., Sole, C. J. and Stone, M. H. (2016). Comparison of methods that assess lower-body stretch-shortening cycle utilization. *J Strength Cond Res, 30*(2), 547-554. doi:10.1519/jsc.0000000000001100

Tornero-Aguilera, J. F., Villegas-Mora, B. E. and Clemente-Suarez, V. J. (2022). Differences in body composition analysis by DEXA, skinfold and BIA methods in young football players. *Children, 9*(11). doi:10.3390/children9111643

Toro-Román, V., Grijota, F. J., Muñoz, D., Maynar-Mariño, M., Clemente-Gil, S. and Robles-Gil, M. C. (2023a). Anthropometry, body composition, and physical fitness in semi-professional soccer players: differences between sexes and playing position. *Applied Sciences, 13*(3). doi:10.3390/app13031249

Toro-Román, V., Munoz, D., Maynar-Marino, M., Clemente-Gil, S. and Robles-Gil, M. C. (2023b). Sex differences in copper concentrations during a sports season in soccer players. *Nutrients, 15*(3). doi:10.3390/nu15030495

Toro-Román, V., Robles-Gil, M. C., Munoz, D., Bartolome, I., Grijota, F. J. and Maynar-Marino, M. (2023c). Sex differences in cadmium and lead concentrations in different biological matrices in athletes. Relationship with iron status. *Environ Toxicol Pharmacol, 99*, 104107. doi:10.1016/j.etap.2023.104107

Toro-Román, V., Robles-Gil, M. C., Munoz, D., Bartolome, I., Siquier-Coll, J. and Maynar-Marino, M. (2022). Extracellular and Intracellular concentrations of molybdenum and zinc in soccer players: sex differences. *Biology, 11*(12). doi:10.3390/biology11121710

Toro-Román, V., Siquier-Coll, J., Grijota Perez, F. J., Maynar-Marino, M., Bartolome-Sanchez, I. and Robles-Gil, M. C. (2024). Plasma, urinary, erythrocyte and platelet zinc concentrations in soccer players. *Nutrients, 16*(16). doi:10.3390/nu16162789

Yilmaz, E., Aydin, T., Kiliç, S. and Toluk, Ö. (2023). Bilateral knee joint isokinetic muscle strength and angle specific balance ratio in soccer players. *Dtsch Z Sportmed, 74*(7), 234-241. doi:10.5960/dzsm.2023.580

Zebis, M. K., Andersen, L. s. L., Ellingsgaard, H. and Aagaard, P. (2011). Rapid hamstring/quadriceps force capacity in male vs. female elite soccer players. *J Strength Cond Res, 25*(7), 1989-1993. doi:10.1519/JSC.0b013e3181e501a6
