# Supplementary material for: Sex differences in association football: a scoping review
Source: PeerJ. 2025 Aug 27;13:e19976. doi: 10.7717/peerj.19976 (PMC12398286; doi:10.7717/peerj.19976)
Supplement: Supplemental Information 4 [file peerj-13-19976-s004.docx]

| **Table S3** Results of the risk of bias assessment. | | | | | | | | |
| --- | --- | --- | --- | --- | --- | --- | --- | --- |
| Author (years) | D1 | D2 | D3 | D4 | D5 | D6 | D7 | D8 |
| Mascherini et al. (2017) | L | L | L | L | L | L | L | L |
| Baker et al. (2020) | H | U | L | L | L | L | L | L |
| Hedt et al. (2022) | L | U | U | L | L | L | L | L |
| Schons et al. (2022) | L | U | L | L | L | L | L | L |
| Tornero-Aguilera et al. (2022) | L | U | L | L | L | L | L | L |
| Toro-Román et al. (2023a) | H | L | L | L | L | L | L | L |
| Ahmad et al. (2024) | L | U | U | L | L | L | L | L |
| Petri et al. (2024a) | L | U | L | L | L | L | L | L |
| Petri et al. (2024b) | L | U | U | L | L | L | L | L |
| Ichinose et al. (1998) | L | U | U | L | L | L | L | L |
| Kanehisa et al. (2003) | L | U | U | L | L | L | L | L |
| Hewett et al. (2006) | L | U | U | L | L | L | L | L |
| Hart et al. (2007) | L | U | U | L | L | L | L | L |
| Brophy et al. (2009) | L | U | U | L | L | L | L | L |
| Zebis et al. (2011) | L | U | U | L | L | L | L | L |
| Burfeind et al. (2012) | L | U | U | L | L | L | L | L |
| Yilmaz et al. (2023) | L | U | L | L | L | L | L | L |
| Steding et al. (2010) | L | U | L | L | L | L | L | L |
| Chamera et al. (2014) | H | U | U | L | L | L | L | L |
| Souglis et al. (2015) | L | U | L | L | L | L | L | L |
| Chamera et al. (2015) | L | U | U | L | L | L | L | L |
| Kostrzewa-Nowak et al. (2015) | L | U | L | L | L | L | L | L |
| Dent et al. (2015) | L | U | U | L | L | L | L | L |
| Sanders et al. (2017) | L | U | U | L | L | L | L | L |
| Souglis et al. (2018) | L | U | L | L | L | L | L | L |
| Sansonio de Morais et al. (2018) | L | U | U | L | L | L | L | L |
| Mascherini et al. (2018) | L | U | L | L | L | L | L | L |
| Magal et al. (2020) | L | U | U | L | L | L | L | L |
| Rodas et al. (2022) | L | U | L | H | L | L | H | L |
| McFadden et al. (2024) | L | U | L | L | L | L | L | L |
| Mujika et al. (2009) | L | U | L | L | L | L | L | L |
| Baumgart et al. (2014) | H | U | L | L | L | L | L | L |
| Suchomel et al. (2015) | L | U | U | L | L | L | L | L |
| McFarland et al. (2016) | L | U | U | L | L | L | L | L |
| Suchomel et al. (2016) | L | U | U | L | L | L | L | L |
| Condello et al. (2016) | L | U | U | L | L | L | L | L |
| Nagano et al. (2016) | L | U | U | L | L | L | L | L |
| Cardoso de Araujo et al. (2018) | H | U | U | L | L | L | H | L |
| Baumgart et al. (2018) | L | U | U | L | L | L | L | L |
| Cardoso de Araujo et al. (2019) | H | U | L | L | L | L | L | L |
| Devismes et al. (2019) | L | U | U | L | L | L | L | L |
| Dolci et al. (2021) | L | U | L | L | L | L | L | L |
| Makaraci et al. (2024) | L | U | L | L | L | L | L | L |
| Putukian et al. (2000) | L | U | U | L | L | L | H | L |

**Table S3** (Continued)

| Author (years) | D1 | D2 | D3 | D4 | D5 | D6 | D7 | D8 |
| --- | --- | --- | --- | --- | --- | --- | --- | --- |
| Barfield et al. (2002) | L | U | U | L | L | L | L | L |
| Orloff et al. (2008) | L | U | U | L | L | L | L | L |
| Gheidi et al. (2010) | L | U | U | L | L | L | L | L |
| Sakamoto et al. (2013) | L | U | U | L | L | L | L | L |
| Bretzin et al. (2016) | L | U | U | L | L | L | L | L |
| Reynolds et al. (2017) | L | U | U | L | L | L | L | L |
| Saunders et al. (2020) | L | U | U | L | L | L | L | L |
| Nelson et al. (2020) | L | U | U | L | L | L | L | L |
| Langdon et al. (2022) | L | U | U | L | L | L | L | L |
| Navandar et al. (2022) | H | U | U | L | L | L | L | L |
| Iitake et al. (2022) | L | U | U | L | L | L | L | L |
| Jackson et al. (2023) | L | U | U | L | L | L | L | L |
| Peek et al. (2024) | L | U | U | L | L | L | L | L |
| Siegle et al. (2012) | L | U | U | L | L | L | L | L |
| Bradley et al. (2014) | L | U | U | L | L | L | L | L |
| Jastrzębski et al. (2017) | H | U | U | L | L | L | L | L |
| McFadden et al. (2020) | L | U | L | L | L | L | L | L |
| Pappalardo et al. (2021) | L | U | U | L | L | L | L | L |
| Coulomb-Cabagno et al. (2006) | L | U | U | L | L | L | L | L |
| Adegbesan (2007) | L | U | U | L | L | L | L | L |
| López-Gajardo et al. (2021) | H | U | L | L | L | L | L | L |
| De la Vega et al. (2022) | H | U | L | L | L | L | L | L |
| Avugos et al. (2022) | H | U | L | L | L | L | L | L |
| Jin et al. (2023) | L | U | U | L | L | L | L | L |
| Bonet et al. (2024) | L | U | U | L | L | L | L | L |
| Ros et al. (2013) | L | U | L | L | L | L | L | L |
| Koikawa et al. (2016) | L | U | U | L | L | L | L | L |
| Gomez-Hixson et al. (2020) | L | U | L | L | L | L | L | L |
| Biggins et al. (2022) | L | U | U | L | L | L | L | L |
| Toro-Román et al. (2022) | H | L | L | L | L | L | L | L |
| Kwon et al. (2023) | L | U | L | L | L | L | L | L |
| Robles-Gil et al. (2023) | H | L | L | L | L | L | L | L |
| Toro-Román et al. (2023b) | H | L | L | L | L | L | L | L |
| Toro-Román et al. (2023c) | H | L | L | L | L | L | L | L |
| Sebastia-Rico et al. (2024) | H | L | L | L | L | L | L | L |
| Toro-Román et al. (2024) | H | L | L | L | L | L | L | L |
| **Abbreviations:** D1, comparability of the target group; D2, target group selection; D3, confounders; D4, measurement of intervention/exposure; D5, blinding of assessors; D6, outcome assessment; D7, incomplete outcome data; D8, selective outcome reporting; L, low risk of bias; U, unclear risk of bias; H, high risk of bias. | | | | | | | | |

**References**

Adegbesan, O. A. (2007). Sources of sport confidence of elite male and female soccer players in Nigeria. *Eur J Sci Res, 18*(2), 217-222.

Ahmad, M. N. and Abu Al Haija, D. M. (2024). Circulating irisin and its connection with indices of body composition in aerobic and anaerobic endurance professional athletes: a case-control study. *Health, sport, rehabilitation, 10*(1), 27-38. doi:10.58962/hsr.2024.10.1.27-38

Avugos, S., Azar, O. H., Sher, E., Gavish, N. and Bar-Eli, M. (2022). Detecting patterns in the behaviour of goalkeepers and kickers in the penalty shootout: a between-gender comparison among score situations. *Int J Sport Exerc Psychol, 21*(2), 196-216. doi:10.1080/1612197x.2022.2066704

Baker, B. S., Chen, Z., Larson, R. D., Bemben, M. G. and Bemben, D. A. (2020). Sex differences in bone density, geometry, and bone strength of competitive soccer players. *J Musculoskelet Neuronal Interact, 20*(1), 62-76.

Barfield, W. R., Kirkendall, D. T. and Yu, B. (2002). Kinematic instep kicking differences between elite female and male soccer players. *J Sport Sci Med, 1*(3), 72-79. doi:10.1097/00005768-200105001-00576

Baumgart, C., Freiwald, J. and Hoppe, M. W. (2018). Sprint mechanical properties of female and different aged male top-level german soccer players. *Sports, 6*(4). doi:10.3390/sports6040161

Baumgart, C., Hoppe, M. W. and Freiwald, J. (2014). Different endurance characteristics of female and male german soccer players. *Biol Sport, 31*(3), 227-232. doi:10.5604/20831862.1111851

Biggins, M., Purtill, H., Fowler, P., O'Sullivan, K. and Cahalan, R. (2022). Impact of long-haul travel to international competition on sleep and recovery in elite male and female soccer athletes. *Int J Sports Physiol Perform, 17*(9), 1361-1370. doi:10.1123/ijspp.2021-0165

Bonet, L., Benito, A., Uso, H., Peraire, M., Haro, G. and Almodovar-Fernandez, I. (2024). Mental health in first- and second-division soccer players: a cross-sectional study. *Sports, 12*(4). doi:10.3390/sports12040106

Bradley, P. S., Dellal, A., Mohr, M., Castellano, J. and Wilkie, A. (2014). Gender differences in match performance characteristics of soccer players competing in the UEFA Champions League. *Hum. Mov. Sci., 33*, 159-171. doi:10.1016/j.humov.2013.07.024

Bretzin, A. C., Mansell, J. L., Tierney, R. T. and McDevitt, J. K. (2016). Sex differences in anthropometrics and heading kinematics among Division I soccer athletes. *Sports Health, 9*(2), 168-173. doi:10.1177/1941738116678615

Brophy, R. H., Chiaia, T. A., Maschi, R., Dodson, C. C., Oh, L. S., Lyman, S., Allen, A. A. and Williams, R. J. (2009). The core and hip in soccer athletes compared by gender. *Int J Sports Med, 30*(9), 663-667. doi:10.1055/s-0029-1225328

Burfeind, K., Hong, J. G. and Stavrianeas, S. (2012). Gender differences in the neuromuscular fitness profiles of NCAA Division III soccer players. *Isokinet Exerc Sci, 20*(2), 115-120. doi:10.3233/ies-2012-0449

Cardoso de Araujo, M., Baumgart, C., Freiwald, J. and Hoppe, M. W. (2019). Contrasts in intermittent endurance performance and heart rate response between female and male soccer players of different playing levels. *Biol Sport, 36*(4), 323-331. doi:10.5114/biolsport.2019.88755

Cardoso de Araujo, M., Baumgart, C., Jansen, C. T., Freiwald, J. and Hoppe, M. W. (2018). Sex differences in physical capacities of German Bundesliga soccer players. *J Strength Cond Res, 34*(8), 2329-2337. doi:10.1519/JSC.0000000000002662

Chamera, T., Spieszny, M., Klocek, T., Kostrzewa-Nowak, D., Nowak, R., Lachowicz, M., Buryta, R. and Cięszczyk, P. (2014). Could biochemical liver profile help to assess metabolic response to aerobic effort in athletes? *J Strength Cond Res, 28*(8), 2180-2186. doi:10.1519/jsc.0000000000000398

Chamera, T., Spieszny, M., Klocek, T., Kostrzewa-Nowak, D., Nowak, R., Lachowicz, M., Buryta, R., Ficek, K., Eider, J., Moska, W. and Cięszczyk, P. (2015). Post-effort changes in activity of traditional diagnostic enzymatic markers in football players' blood. *J Med Biochem, 34*(2), 179-190. doi:10.2478/jomb-2014-0035

Condello, G., Kernozek, T. W., Tessitore, A. and Foster, C. (2016). Biomechanical analysis of a change-of-direction task in collegiate soccer players. *Int J Sports Physiol Perform, 11*(1), 96-101. doi:10.1123/ijspp.2014-0458

Coulomb‐Cabagno, G. and Rascle, O. (2006). Team sports players' observed aggression as a function of gender, competitive level, and sport type. *Journal of Applied Social Psychology, 36*(8), 1980-2000. doi:10.1111/j.0021-9029.2006.00090.x

De la Vega, R., Gómez, J., Vaquero-Cristobal, R., Horcajo, J. and Abenza-Cano, L. (2022). Objective comparison of achievement motivation and competitiveness among semi-professional male and female football players. *Sustainability, 14*(9). doi:10.3390/su14095258

Dent, J. R., Edge, J. A., Hawke, E., McMahon, C. and Mündel, T. (2015). Sex differences in acute translational repressor 4E-BP1 activity and sprint performance in response to repeated-sprint exercise in team sport athletes. *J Sci Med Sport, 18*(6), 730-736. doi:10.1016/j.jsams.2014.10.006

Devismes, M., Aeles, J., Philips, J. and Vanwanseele, B. (2019). Sprint force-velocity profiles in soccer players: impact of sex and playing level. *Sports Biomech, 20*(8), 947-957. doi:10.1080/14763141.2019.1618900

Dolci, F., Kilding, A., Spiteri, T., Chivers, P., Piggott, B., Maiorana, A. and Hart, N. H. (2021). Characterising running economy and change of direction economy between soccer players of different playing positions, levels and sex. *Eur J Sport Sci, 22*(8), 1167-1176. doi:10.1080/17461391.2021.1953151

Gomez-Hixson, K., Biagioni, E. and Brown, M. L. (2020). Significant differences in dietary intake of NCAA Division III soccer players compared to recommended levels. *J Am Coll Health, 70*(1), 150-157. doi:10.1080/07448481.2020.1728279

Hart, J. M., Garrison, J. C., Kerrigan, D. C., Palmieri-Smith, R. and Ingersoll, C. D. (2007). Gender differences in gluteus medius muscle activity exist in soccer players performing a forward jump. *Res Sports Med, 15*(2), 147-155. doi:10.1080/15438620701405289

Hedt, C. A., Le, J. T., Heimdal, T., Vickery, J., Orozco, E., McCulloch, P. C. and Lambert, B. S. (2022). Sex-related anthropometrics in a lower-body mobility assessment among professional soccer athletes. *Int J Sports Phys Ther, 17*(3), 474-482. doi:10.26603/001c.32595

Hewett, T. E., Ford, K. R., Myer, G. D., Wanstrath, K. and Scheper, M. (2006). Gender differences in hip adduction motion and torque during a single-leg agility maneuver. *J Orthop Res, 24*(3), 416-421. doi:10.1002/jor.20056

Ichinose, Y., Kanehisa, H., Ito, M., Kawakami, Y. and Fukunaga, T. (1998). Morphological and functional differences in the elbow extensor muscle between highly trained male and female athletes. *Eur J Appl Physiol Occup Physiol, 78*(2), 109-114. doi:10.1007/s004210050394

Iitake, T., Hioki, M., Takahashi, H. and Nunome, H. (2022). Sex difference in soccer instep kicking. *J Sports Sci, 40*(20), 2217-2224. doi:10.1080/02640414.2022.2139881

Jackson, B. C., Rogerson, C. E., Bradney, D. A., Breedlove, K. M. and Bowman, T. G. (2023). Preparedness during head impacts in intercollegiate men’s and women’s soccer athletes. *Biomechanics, 3*(1), 45-51. doi:10.3390/biomechanics3010004

Jastrzębski, Z. and Radzimiński, Ł. (2017). Default and individual comparison of physiological responses and time-motion analysis in male and female soccer players during small-sided games. *J Hum Sport Exerc, 12*(4), 1176-1185. doi:10.14198/jhse.2017.124.04

Jin, P., Ji, Z., Wang, T. and Zhu, X. (2023). Association between sports expertise and visual attention in male and female soccer players. *PeerJ, 11*, e16286. doi:10.7717/peerj.16286

Kanehisa, H., Muraoka, Y., Kawakami, Y. and Fukunaga, T. (2003). Fascicle arrangements of vastus lateralis and gastrocnemius muscles in highly trained soccer players and swimmers of both genders. *Int J Sports Med, 24*(2), 90-95. doi:10.1055/s-2003-38197

Koikawa, N., Shimada, S., Suda, S., Murata, A. and Kasai, T. (2016). Sex differences in subjective sleep quality, sleepiness, and health-related quality of life among collegiate soccer players. *Sleep Biol Rhythms, 14*(4), 377-386. doi:10.1007/s41105-016-0068-4

Kostrzewa-Nowak, D., Nowak, R., Chamera, T., Buryta, R., Moska, W. and Cięszczyk, P. (2015). Post-effort chances in C-reactive protein level among soccer players at the end of the training season. *J Strength Cond Res, 29*(5), 1399-1405. doi:10.1519/jsc.0000000000000753

Kwon, J., Nishisaka, M. M., McGrath, A. F., Kristo, A. S., Sikalidis, A. K. and Reaves, S. K. (2023). Protein intake in NCAA Division 1 soccer players: assessment of daily amounts, distribution patterns, and leucine levels as a quality indicator. *Sports, 11*(2). doi:10.3390/sports11020045

Langdon, S., Goedhart, E., Oosterlaan, J. and Konigs, M. (2022). Heading exposure in elite football (soccer): a study in adolescent, young adult, and adult male and female players. *Med Sci Sports Exerc, 54*(9), 1459-1465. doi:10.1249/MSS.0000000000002945

López-Gajardo, M. Á., Ponce-Bordón, J. C., Rubio-Morales, A., Llanos-Muñoz, R. and Díaz-García, J. (2021). The role of perceived justice on satisfaction with the coach: gender differences in a longitudinal study. *Sustainability, 14*(1). doi:10.3390/su14010401

Magal, M., Liette, N. C., Crowley, S. K., Hoffman, J. R. and Thomas, K. S. (2020). Sex-based performance responses to an acute sprint interval cycling training session in collegiate athletes. *Res Q Exerc Sport, 92*(3), 469-476. doi:10.1080/02701367.2020.1751026

Makaraci, Y., Nas, K., GÜNdÜZ, K. and İLeri, M. (2024). Relationship between functional movement screen scores and postural stability in football players: an asymmetrical approach. *Balt J Health Phys Act, 16*(1), Article6-Article6. doi:10.29359/bjhpa.16.1.06

Mascherini, G., Castizo-Olier, J., Irurtia, A., Petri, C. and Galanti, G. (2017). Differences between the sexes in athletes' body composition and lower limb bioimpedance values. *Muscles, Ligaments Tendons J, 7*(4), 573-581. doi:10.11138/mltj/2017.7.4.573

Mascherini, G., Petri, C. and Galanti, G. (2018). Link between body cellular mass and left ventricular hypertrophy in female and male athletes. *J Sports Med Phys Fitness, 59*(1), 164-170. doi:10.23736/S0022-4707.18.08259-2

McFadden, B. A., Walker, A. J., Bozzini, B. N., Sanders, D. J. and Arent, S. M. (2020). Comparison of internal and external training loads in male and female collegiate soccer players during practices vs. games. *J Strength Cond Res, 34*(4), 969-974. doi:10.1519/JSC.0000000000003485

McFadden, B. A., Walker, A. J., Cintineo, H. P., Bozzini, B. N., Sanders, D. J., Chandler, A. J. and Arent, S. M. (2024). Sex differences in physiological responses to a national collegiate athletic association Division I soccer season. *J Strength Cond Res*. doi:10.1519/JSC.0000000000004882

McFarland, I. T., Dawes, J. J., Elder, C. L. and Lockie, R. G. (2016). Relationship of two vertical jumping tests to sprint and change of direction speed among male and female collegiate soccer players. *Sports, 4*(1). doi:10.3390/sports4010011

Mujika, I., Santisteban, J., Impellizzeri, F. M. and Castagna, C. (2009). Fitness determinants of success in men's and women's football. *J Sports Sci, 27*(2), 107-114. doi:10.1080/02640410802428071

Gheidi, N. and Sadeghi, H. (2010). Kinematic comparison of successful and unsuccessful instep kick in indoor soccer. *Am J Applied Sci, 7*(10), 1334-1340. doi:10.3844/ajassp.2010.1334.1340

Nagano, Y., Sasaki, S., Higashihara, A. and Ishii, H. (2016). Gender differences in trunk acceleration and related posture during shuttle run cutting. *Int Biomech, 3*(1), 33-39. doi:10.1080/23335432.2016.1191372

Navandar, A., Kipp, K. and Navarro, E. (2022). Hip and knee joint angle patterns and kicking velocity in female and male professional soccer players: a principal component analysis of waveforms approach. *J Sports Sci, 40*(17), 1919-1930. doi:10.1080/02640414.2022.2121022

Nelson, K. M., Daidone, E. H. K., Breedlove, K. M., Bradney, D. A. and Bowman, T. G. (2020). Head impact characteristics based on player position in collegiate soccer athletes. *Int J Athl Ther Trai, 26*(2), 111-115. doi:10.1123/ijatt.2019-0095

Orloff, H., Sumida, B., Chow, J., Habibi, L., Fujino, A. and Kramer, B. (2008). Ground reaction forces and kinematics of plant leg position during instep kicking in male and female collegiate soccer players. *Sports Biomech, 7*(2), 238-247. doi:10.1080/14763140701841704

Pappalardo, L., Rossi, A., Natilli, M. and Cintia, P. (2021). Explaining the difference between men's and women's football. *PLoS one, 16*(8), e0255407. doi:10.1371/journal.pone.0255407

Peek, K., Georgieva, J., Serner, A. and Orest, F. (2024). Differences in the technical performance of heading between men and women football players during FIFA World Cup 2022 and FIFA Women's World Cup 2023 matches. *BMJ Open Sport Exerc Med, 10*(3), e002066. doi:10.1136/bmjsem-2024-002066

Petri, C., Campa, F., Holway, F., Pengue, L. and Arrones, L. S. (2024a). ISAK-based anthropometric standards for elite male and female soccer players. *Sports, 12*(3). doi:10.3390/sports12030069

Petri, C., Pengue, L., Bartolini, A., Pistolesi, D. and Arrones, L. S. (2024b). Body composition changes in male and female elite soccer players: effects of a nutritional program led by a sport nutritionist. *Nutrients, 16*(3). doi:10.3390/nu16030334

Putukian, M., Echemendia, R. J. and Mackin, S. (2000). The acute neuropsychological effects of heading in soccer: a pilot study. *Clin J Sport Med, 10*(2), 104-109. doi:10.1097/00042752-200004000-00004

Reynolds, B. B., Patrie, J., Henry, E. J., Goodkin, H. P., Broshek, D. K., Wintermark, M. and Druzgal, T. J. (2017). Effects of sex and event type on head impact in collegiate soccer. *Orthop J Sports Med, 5*(4), 2325967117701708. doi:10.1177/2325967117701708

Robles-Gil, M. C., Toro-Roman, V., Maynar-Marino, M., Siquier-Coll, J., Bartolome, I. and Grijota, F. J. (2023). Aluminum concentrations in male and female football players during the season. *Toxics, 11*(11). doi:10.3390/toxics11110920

Rodas, G., Ferrer, E., Reche, X., Sanjuan-Herraez, J. D., McCall, A. and Quintas, G. (2022). A targeted metabolic analysis of football players and its association to player load: comparison between women and men profiles. *Front Physiol, 13*, 923608. doi:10.3389/fphys.2022.923608

Ros, A. G., Holm, S. E., Fridén, C. and Heijne, A. I. (2013). Responsiveness of the one-leg hop test and the square hop test to fatiguing intermittent aerobic work and subsequent recovery. *J Strength Cond Res, 27*(4), 988-994. doi:10.1519/JSC.0b013e31825feb5b

Sakamoto, K. and Asai, T. (2013). Comparison of kicking motion characteristics at ball impact between female and male soccer players. *Int J Sports Sci Coa, 8*(1), 63-76. doi:10.1260/1747-9541.8.1.63

Sanders, G. J., Turner, Z., Boos, B., Peacock, C. A., Peveler, W. and Lipping, A. (2017). Aerobic capacity is related to repeated sprint ability with sprint distances less than 40 meters. *Int J Exerc Sci, 10*(2), 197-204. doi: 10.70252/gcdn2088

Sansonio de Morais, A., Ferreira, G. A., Lima-Silva, A. E. and Gomes Filho, A. (2018). Gender-related cardiac dimension differences between female and male professional soccer players. *J Sports Med Phys Fitness, 58*(9), 1354-1359. doi:10.23736/S0022-4707.17.07422-9

Saunders, T. D., Le, R. K., Breedlove, K. M., Bradney, D. A. and Bowman, T. G. (2020). Sex differences in mechanisms of head impacts in collegiate soccer athletes. *Clin Biomech, 74*, 14-20. doi:10.1016/j.clinbiomech.2020.02.003

Schons, P., Birk Preissler, A. A., Oliveira, R., Brito, J. P., Clemente, F. M., Droescher de Vargas, G., Moraes Klein, L. and Kruel, L. F. M. (2022). Comparisons and correlations between the anthropometric profile and physical performance of professional female and male soccer players: Individualities that should be considered in training. *Int J Sports Sci Coa, 18*(6), 2004-2014. doi:10.1177/17479541221131649

Sebastia-Rico, J., Soriano, J. M., Sanchis-Chorda, J., Garcia-Fernandez, A. F., Lopez-Mateu, P., de la Cruz Marcos, S. and Martinez-Sanz, J. M. (2024). Analysis of fluid balance and urine values in elite soccer players: impact of different environments, playing positions, sexes, and competitive levels. *Nutrients, 16*(6). doi:10.3390/nu16060903

Siegle, M. and Lames, M. (2012). Influences on frequency and duration of game stoppages during soccer. *Int J Perform Anal Spor, 12*(1), 101-111. doi:10.1080/24748668.2012.11868586

Souglis, A., Bogdanis, G. C., Chryssanthopoulos, C., Apostolidis, N. and Geladas, N. D. (2018). Time course of oxidative stress, inflammation, and muscle damage markers for 5 days after a soccer match: effects of sex and playing position. *J Strength Cond Res, 32*(7), 2045-2054. doi:10.1519/JSC.0000000000002436

Souglis, A., Papapanagiotou, A., Bogdanis, G. C., Travlos, A. K., Apostolidis, N. G. and Geladas, N. D. (2015). Comparison of inflammatory responses to a soccer match between elite male and female players. *J Strength Cond Res, 29*(5), 1227-1233. doi:10.1519/jsc.0000000000000767

Steding, K., Engblom, H., Buhre, T., Carlsson, M., Mosén, H., Wohlfart, B. and Arheden, H. (2010). Relation between cardiac dimensions and peak oxygen uptake. *J Cardiovasc Magn Reson, 12*(1), 8. doi:10.1186/1532-429X-12-8

Suchomel, T. J., Sole, C. J., Bailey, C. A., Grazer, J. L. and Beckham, G. K. (2015). A comparison of reactive strength index-modified between six U.S. collegiate athletic teams. *J Strength Cond Res, 29*(5), 1310-1316. doi:10.1519/jsc.0000000000000761

Suchomel, T. J., Sole, C. J. and Stone, M. H. (2016). Comparison of methods that assess lower-body stretch-shortening cycle utilization. *J Strength Cond Res, 30*(2), 547-554. doi:10.1519/jsc.0000000000001100

Tornero-Aguilera, J. F., Villegas-Mora, B. E. and Clemente-Suarez, V. J. (2022). Differences in body composition analysis by DEXA, skinfold and BIA methods in young football players. *Children, 9*(11). doi:10.3390/children9111643

Toro-Román, V., Grijota, F. J., Muñoz, D., Maynar-Mariño, M., Clemente-Gil, S. and Robles-Gil, M. C. (2023a). Anthropometry, body composition, and physical fitness in semi-professional soccer players: differences between sexes and playing position. *Applied Sciences, 13*(3). doi:10.3390/app13031249

Toro-Román, V., Munoz, D., Maynar-Marino, M., Clemente-Gil, S. and Robles-Gil, M. C. (2023b). Sex differences in copper concentrations during a sports season in soccer players. *Nutrients, 15*(3). doi:10.3390/nu15030495

Toro-Román, V., Robles-Gil, M. C., Munoz, D., Bartolome, I., Grijota, F. J. and Maynar-Marino, M. (2023c). Sex differences in cadmium and lead concentrations in different biological matrices in athletes. Relationship with iron status. *Environ Toxicol Pharmacol, 99*, 104107. doi:10.1016/j.etap.2023.104107

Toro-Román, V., Robles-Gil, M. C., Munoz, D., Bartolome, I., Siquier-Coll, J. and Maynar-Marino, M. (2022). Extracellular and Intracellular concentrations of molybdenum and zinc in soccer players: sex differences. *Biology, 11*(12). doi:10.3390/biology11121710

Toro-Román, V., Siquier-Coll, J., Grijota Perez, F. J., Maynar-Marino, M., Bartolome-Sanchez, I. and Robles-Gil, M. C. (2024). Plasma, urinary, erythrocyte and platelet zinc concentrations in soccer players. *Nutrients, 16*(16). doi:10.3390/nu16162789

Yilmaz, E., Aydin, T., Kiliç, S. and Toluk, Ö. (2023). Bilateral knee joint isokinetic muscle strength and angle specific balance ratio in soccer players. *Dtsch Z Sportmed, 74*(7), 234-241. doi:10.5960/dzsm.2023.580

Zebis, M. K., Andersen, L. s. L., Ellingsgaard, H. and Aagaard, P. (2011). Rapid hamstring/quadriceps force capacity in male vs. female elite soccer players. *J Strength Cond Res, 25*(7), 1989-1993. doi:10.1519/JSC.0b013e3181e501a6
